# Endoscopic Findings in Loin Pain Hematuria Syndrome: Concentric Clot in Calyceal Fornices

**DOI:** 10.1155/2008/721850

**Published:** 2009-02-16

**Authors:** Benjamin K. Canales, Andrew Windsperger, Stephen Lukasewycz, Manoj Monga

**Affiliations:** Department of Urologic Surgery, University of Minnesota, Minneapolis, MN 55455-0392, USA

## Abstract

The loin pain hematuria syndrome (LPHS) creates a considerable burden, both for patients afflicted with the disease and for those involved in medical management and diagnosis. To date, the diagnosis of LPHS remains one of exclusion, with some speculation regarding the extent of actual pathology. We report ureteroscopic findings in 2 cases of LPHS. These findings provide objective confirmation of underlying pathology in a difficult-to-manage disease process.

## 1. INTRODUCTION

Loin pain hematuria syndrome (LPHS) describes a constellation of clinical features including recurrent flank pain and intermittent hematuria without a readily identifiable cause [1]. Historically, LPHS has been associated with psychiatric illness and is now widely considered a type of somatoform pain disorder [2, 3]. Multidisciplinary pain management strategies include analgesics, nerve blockade, renal denervation, and/or nephrectomy with autotransplantation [4]. Though some studies suggest that long-term prognosis is usually excellent [4], many urologists and patients would contest this.

## 2. METHODS

Two patients with severe chronic flank pain punctuated by intermittent gross hematuria were evaluated after laboratory and radiographic evaluation failed to delineate a recognizable pathology, raising the suspicion of a diagnosis of LPHS. Patients underwent ureteroscopic evaluation using a no-touch technique. Ureteroscopic evaluation was performed on the side where the patient reported pain, and/or bloody efflux was noted from the ureteral orifice.

After informed consent and induction of general anesthesia, visual inspection of the bladder was performed using a 19 Fr Rigid cystoscope. Subsequently, a 6/7.5 Fr Wolf semirigid ureteroscope was inserted and the ureteral orifice of the symptomatic side cannulated without the use of a guidewire. The ureteroscope was advanced under direct vision with the use of minimal hand irrigation of saline, utilizing a Boston Scientific single-action pump. Semirigid ureteroscopy was utilized to the level of the proximal ureter. A Boston Scientific sensor guidewire (0.035”) was advanced through the working channel to the tip of the ureteroscope and the ureteroscope was then withdrawn, using intermittent fluoroscopy to confirm that the guidewire did not advance beyond the point of ureteroscopic inspection. The Olympus URF-P3 flexible ureteroscope was then backloaded over the floppy-tip of the sensor wire and advanced under fluoroscopic guidance to the point of last inspection, following which the guidewire was removed. Systematic inspection of the entire collecting system was then performed with the flexible ureteroscope, taking care to utilize minimal irrigation throughout the procedure. A Holmium laser was kept on stand-by in the event that pathology was to be identified and could be successfully ablated. Following completion of the procedure, the ureter was inspected as the ureteroscope was withdrawn to evaluate for injury, and as none was identified a ureteral stent was not left in place.

## 3. RESULTS

Patient 1 is a 26-year-old female with a 3-year history of severe right flank pain and gross hematuria beginning in 2003. Her pain was described as stabbing and located in the lower back and right flank with radiation to the lower abdomen. She reported episodes of intense pain every three to four months that lasted 2–4 weeks, with a notable basal level of pain in between exacerbations. The patient underwent ureteral stent placement and ureteroscopic extraction of a small stone following a bout of pain in 2004, and following this, had CT scans with and without contrast that did not demonstrate any stones, masses, or hydronephrosis. The patient presented in early 2005 with a recurrence of symptoms including pain, fever, and microscopic hematuria. A CT scan with and without IV contrast obtained at this time did not demonstrate any evidence of renal calculi, hydronephrosis, or renal mass. With normal imaging studies, the patient was treated with IV antibiotics with eventual relief of symptoms. In late 2005, the patient was admitted to the hospital with severe right flank pain, fever, and gross hematuria. Her urine specimen contained large blood without organisms, signs of infection, or positive culture or cytology. A nephrology consult was obtained for a nephritis workup revealing a normal serum complement and ANCA screen, a negative Hepatitis C antibody, and negative antiglomerular basement antibody test. Further studies included an unremarkable CT angiogram of the kidney to evaluate for AV malformation. The decision then made to perform bilateral diagnostic ureteroscopy during this episode of pain and gross hematuria. Right rigid and flexible ureteroscopy was performed, using a wireless, no-touch technique with minimal irrigation fluid. Diffuse bleeding in a pattern of ring-like clots was noted from each fornix surrounding all calyces of the collecting system, without any identifiable masses or neoplasia (see Supplementary Video in Supplementary Material available online at doi:10.1155/2009/721850). The patient received an inpatient pain management consultation and was maintained on Neurontin 300 mg three times daily and Dilaudid 4–8 mg every four to six hours. The patient is currently seeking counseling regarding more aggressive treatments such as nephrectomy or autotransplantation.

Patient 2 is an 18-year-old female with a 2-year history of severe left flank pain with intermittent gross hematuria. The patient presented in 2005 to an outside institution and underwent ureteroscopic evaluation for her flank pain. A small hemangioma was reportedly identified and cauterized during this evaluation. Additionally, the patient was noted to have low-grade vesicoureteral reflux, and a deflux procedure was performed in 2006 with alleviation of pain associated with voiding. However, pain not associated with voiding persisted, and the patient underwent laparoscopic evaluation by gynecology in 2006. This showed the presence of a Meckel's diverticulum and several small spots of endometriosis, and the patient underwent diverticulectomy, interval appendectomy, and fulguration of the endometriosis. The patient continued to have intermittent bouts of severe left flank pain, often requiring ER visits and significant amounts of IV narcotics. The patient underwent several CT scans with and without IV contrast that did not demonstrate any evidence of renal calculi, masses, or hydronephrosis.

An outpatient renal consult was requested, and a full unremarkable work up for her hematuria was performed, including urine microscopy and cytology, CT angiogram, 24-hour urine collection studies, and negative nephritis work up. She then underwent left ureteroscopic evaluation utilizing the wireless, no-touch technique, which again demonstrated a similar pattern of diffuse bleeding in a pattern of ring-like clots from each fornix surrounding all calyces of the collecting system of the left kidney. The patient received an inpatient pain management consultation and was maintained on Levsin 0.25 mg every 24 hours, a 50 mcg Fentanyl patch every 72 hours, Dilaudid 8 mg every four hours, Neurontin 300 mg three times daily, and Flexeril 10 mg three times daily. The patient eventually elected to undergo left-sided nephrectomy with autotransplantation into the right lower quadrant of her abdomen with resolution of her symptoms. The patient has since developed right flank pain suggestive of LPHS in her contralateral kidney, and has undergone intrathecal catheter placement, epidural catheter placement, and most recently a right lower quandrant fascial pain block.

## 4. DISCUSSION

Though some studies suggest that long-term prognosis for LPHS is excellent [4], many urologists and LPHS patients would contest this. Rates of spontaneous resolution of pain symptoms in LPHS have been reported to typically occur over a 2–5-year period [5]. For those without pain resolution, prognosis can be poor and treatment may involve a multidisciplinary approach with both pain management services (chronic analgesics, nerve blockade, open or laparoscopic renal denervation, and/or nephrectomy with autotransplantation [4]) and psychiatric evaluation (LPHS is considered by some to be a type of somatoform pain disorder [2, 3]).

**Figure 1:**
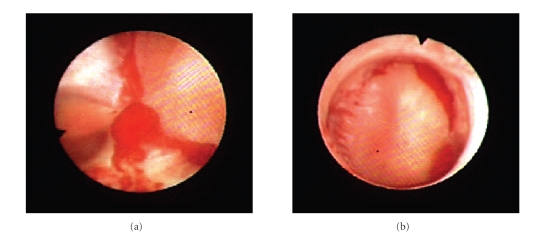
Ureteroscopic images demonstrating ring-like clots surrounding each calyx in the collecting system.

The pathogenesis of flank pain associated with LPHS is thought to be due to glomerular capillary hemorrhage resulting in tubular obstruction and interstitial edema [6]. Over time, chronic edema is thought to result in capsular distention, pain, and further tubular obstruction and hemorrhage. To our knowledge, we are the first to report the endoscopic findings of LPHS. Both LPHS patients underwent diagnostic ureteroscopy using minimal irrigation fluid (to avoid distension of the renal pelvis) and a wireless, no-touch technique (to rule out iatrogenic, traumatic cause of bleeding). In both patients, ring-like clots consistent with recent hemorrhage were identified from each fornix surrounding all calyces of the collecting system (Figures 1(a) and 1(b)). Because diagnosis is the first step in understanding pathophysiology, we hypothesize that these endoscopic findings may represent a clinical, endoscopic manifestation of glomerular hemorrhage and obstruction that has not been previously reported.

It is important to realize that both LPHS patients underwent unremarkable full work-up, with CT urogram, CT angiography, nephrology consultation, and laboratory investigation. All other treatable causes of pain and hematuria must be ruled out in these cases. Once excluded, however, upper tract endoscopy demonstrating hematuria and concentric calyceal clots may strengthen the diagnosis of LPHS and confirm the presence of a physiological disorder in this poorly understood disease. This objective confirmation of disease may result in more definitive diagnoses of LPHS, though a larger series is needed to confirm the results. Such supportive evidence is critical as more aggressive pain management and/or surgical approaches are considered by the patient and family.

## Supplementary Material

The Supplementary Video shows diffuse bleeding in a pattern of ring-like clots that was noted from each fornix surrounding all calyces of the collecting system, without any identifiable masses or neoplasia.
